# Generated stresses finite element analysis of restored endodontically treated molar using single versus double titanium posts: Abbreviated title: generated stresses finite element analysis of restored endodontically treated molar

**DOI:** 10.1038/s41598-025-26931-z

**Published:** 2025-11-28

**Authors:** Mai Soliman, Amal Abdallah A. Abo-Elmagd, Elzahraa Eldwakhly, Alhanoof Aldegheishem, Manal M. Abdelhafeez

**Affiliations:** 1 Department of Clinical Dental Sciences, College of Dentistry, Princess Nourah bint Abdulrahman University, P.O. Box 84428, Riyadh, 11671 Saudi Arabia; 2 Department of Substitute Dental Sciences, College of Dentistry and Pharmacy, Buraydah Private Colleges, P.O. Box 31717, Buraydah, 51418 Saudi Arabia; 3 Department of Fixed Prosthodontics, Faculty of Dental Surgery, Misr University for Science and Technology (MUST), 6th of October City, P.O. Box 77, Giza, 12566 Egypt; 4 Department of Conservative Dental Sciences, College of Dentistry, Qassim University, P.O. Box 1162, Buraydah, 51452 Saudi Arabia

**Keywords:** Finite element analysis, Dental restoration, Endodontically treated teeth, Titanium posts, Stress distribution, Fixed prosthodontics, Root canal treatment

## Abstract

When teeth are extensively injured, the restoration of Endodontically Treated Teeth (ETT) presents substantial obstacles. For these restorations to be successful over the long term, it is essential to ensure appropriate retention and resistance to fracture. The purpose of this study is to use 3D finite element analysis (FEA) to assess the stress distribution in severely damaged ETT following the installation of single versus double titanium posts. Cone beam computed tomography (CBCT) was used to scan an undamaged human mandibular first molar. Three post designs were examined; Model D received one distal post, Model DMB had two posts; distal and mesiobuccal posts, and Model DML included two posts; distal and mesiolingual posts. FEA was used to examine stress distribution and evaluate maximum stress at six areas: occlusal surface, finish line, furcation area, and along the root canal length at 7 mm, 5 mm, and 3 mm from the apex. Model D recorded the highest stress values at all assessed locations, while Model DML reported the least stress values. Model DMB showed intermediate levels of stress. When compared to a single post, the use of two posts considerably reduced stress concentrations, especially when inserted in the distal and mesiolingual canals. FEA proved to be a useful tool in evaluating and directing clinical decisions for repairing ETT. Occlusal surface followed by finish line locations reported the highest stress values in all tested models.

**Supplementary Information:**

The online version contains supplementary material available at 10.1038/s41598-025-26931-z.

## Introduction

Restoration of badly destructed brittle endodontically treated teeth (ETT) is one of the most common challenges in the dental field as several factors influence treatment success such as the amount of structural loss, tooth position, occlusal forces, periodontal condition of the teeth, and esthetic concerns. Thickness of the remaining sound dentine walls and cusps are the main features which establish the restorative materials and the procedures selected for restoration of endodontically treated teeth^1,2^.

The loss of tooth structure caused by extensive tissue loss, the loss of moisture content and flexibility, and endodontic treatment procedure itself are responsible for reduced tooth resistance, which increases susceptibility to fracture^3^. Moreover, posts are indicated in case of major coronal tooth structure loss to aid in the retention of the core material and to distribute forces along the tooth to reduce the risk of fracture. The resistance of the endodontically treated tooth to fracture is greatly influenced by variable materials available for both the post and core with variant elastic modulus. In addition, post diameter, height, and either posts are prefabricated or custom-made contribute to stress distribution in endodontically treated teeth^4^.

As fracture and loosening of post and core systems were reported the most common failure of endodontically treated teeth, several studies aimed to assess different restorative techniques that could reinforce the remaining dental structure to minimize the stress concentration in the remaining tooth structure and the probability of fracture through alternative restorative procedures^5,6^.

Coelho. CS et al.^7^ found that the use of custom cast posts, stainless steel posts, zirconia posts, and titanium posts resulted in increased stress in the post itself when compared to glass fiber posts and carbon fiber posts. Similarly, Nokar S et al.^8^ reported that stainless steel and titanium posts showed lower stresses in dentin compared to fiber reinforced composite posts.

Several independent factors, such as the design, surface texture, diameter, and length of the posts, determine the long-term clinical service of prefabricated posts^9^. FILPOST Endodontic Root Canal Post (Filhol Dental, Leamington Spa, U.K.) is a prefabricated post that consists of 99.8% pure titanium which is biocompatible material showing compatibility with all dental materials. It will not corrode, and it can be customized to suit the canal anatomy without risk of fracture that enabling easy insertion of multiple posts into converging canals^10^.

First permanent molars in young patients are particularly susceptible to severe decay and early extraction due to their early eruption and insufficient cultural health awareness. These teeth often lack adequate structure to retain restorations, increasing their fracture risk and leading to permanent loss of function^11,12^.

In case of restoration of endodontically treated severely damaged posterior teeth, there is limited evidence if using more than one post is necessary for better stress distribution that will enhance structural durability and mechanical success. The use of two posts in such cases is not widely discussed^13^. Recently, a study by Spicciarelli V et al.^14^ reported that the fracture strength of teeth restored with a single post cemented in the palatal root canal was less than teeth restored with two posts in palatal and buccal root canals.

Zhong et al. (2024) conducted a similarly structured FEA on a maxillary first molar with a 4-wall defect and ferrule, comparing models with one post (e.g., palatal alone) versus combinations like palatal + mesiobuccal or palatal + distobuccal, and concluded that the positioning and number of posts significantly affected stress distribution and had direct implications for restoration design and fracture risk^15^.

Measuring stress concentrations accurately on different tooth surfaces is vital for selecting the most suitable retention methods with minimal adverse effects on ETT. FEA is a highly effective method for this purpose. It uses computerized numerical techniques to calculate the strength and behavior of structures by breaking them down into numerous finite elements connected by complex system of points called nodes which make a grid called a mesh that simulates material and structural properties which define how the structure will react to certain loading conditions^16^. The mesh acts like a spider web and from each node there extends a mesh element to each of the adjacent nodes. Once the geometry, materials, and boundary conditions are set, the next step is to run the FEA software to obtain a physical displacement at each node. The strain data that is observed is then used to compute the stress data at each node. A graphical postprocessor is then used to process all of this data and display it superimposed over the geometry model of the part with color coded stress^17^.

FEA’s precision and accuracy make it invaluable in dental research, particularly for simulating biophysical phenomena in in computerized models for teeth and their supporting structures^18^. The finite element method is considered to be an extremely useful tool to simulate the mechanical effects of chewing forces acting on the periodontal ligament (PDL) and on the dental hard tissues^17^.

Limited previous studies evaluated and compared the effect of using of single versus double titanium posts on the stresses generated on endodontically treated molars. Therefore, the purpose of the present in vitro study using FEA is to assess stress distribution and the maximum stress value exerted on EET mandibular first molar when restored with single post inserted in the distal canal versus double-posts inserted in distal canal in conjunction with mesiobuccal or mesiolingual canal, along six locations along the crown and the root.

## Materials and methods

### Model generation

**Fig. 1:**
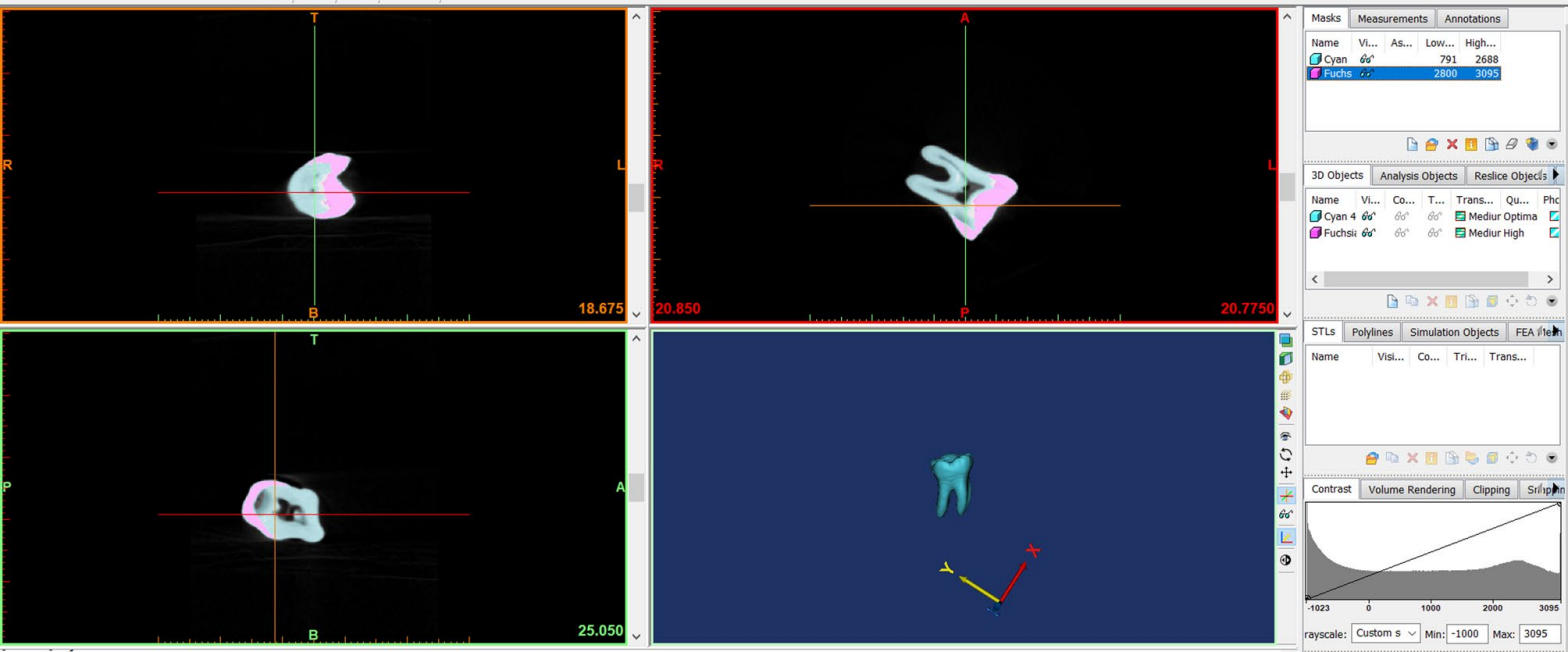
Processed Image of mandibular first molar using the materialize interactive medical image control system.

This in-vitro study granted ethical approval from the ethics committee the Deanship of Scientific Research, Princess Nourah bint Abdulrahman University under the study number IRB (23–0628). All methods were performed in accordance with the relevant guidelines and regulations. A fully developed human right mandibular first molar was scanned using high-resolution cone beam computed tomography (CBCT) (Planmeca ProMax 3D MID; Planmeca, Helsinki, Finland) operating at 90 kV and 12 mA with a voxel dimension of 75 μm, generating a total of 668 images. The images were processed using the Materialize Interactive Medical Image Control System (MIMICS 19.0; Materialise, Leuven, Belgium) to identify enamel and dentin tissues using voxel intensity and the mathematical region growing feature (Fig. 1). A three-dimensional stereolithography (3D-STL) file format (Provided as Supplementary Material) was then exported for both enamel and dentin structures, and the data was optimized using the 3-Matic Medical 11.0 software (Materialise NV).

**Fig. 2:**
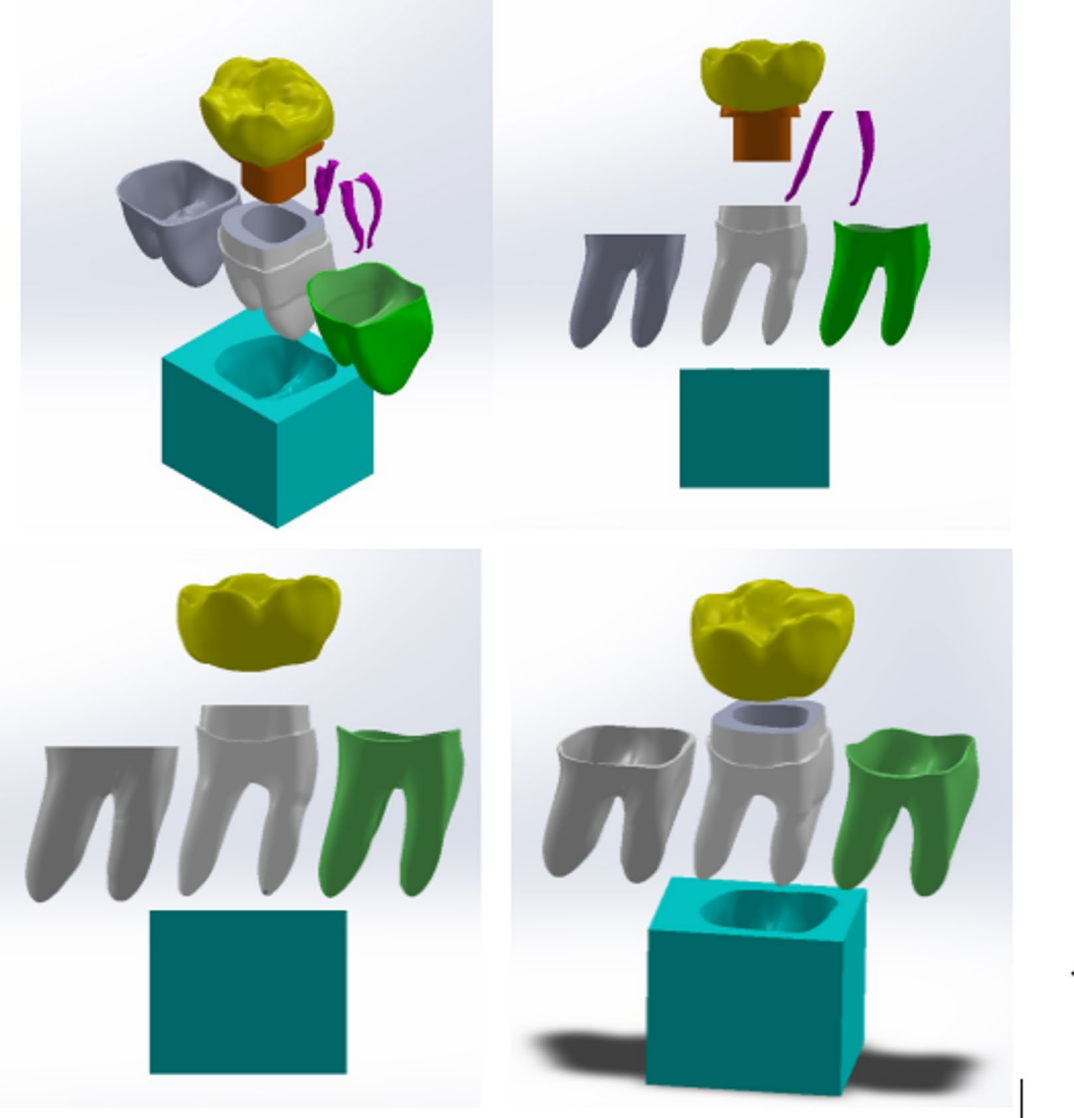
3D model of mandibular first molar components; all-ceramic crown, composite core, dentine, root canals,  cementum, periodontal ligament,  bone.

The file was imported into SolidWorks software 2018 (Dassault Systems, Cedex, France), where enamel and dentin were combined. The surrounding periodontal ligaments (PDL) were then established, extending from the cementoenamel junction (CEJ) to the apical portion of the root with a uniform thickness of 200 μm, and supported by alveolar bone 3 mm below the CEJ (Fig. 2).

### Model preparation

**Fig. 3:**
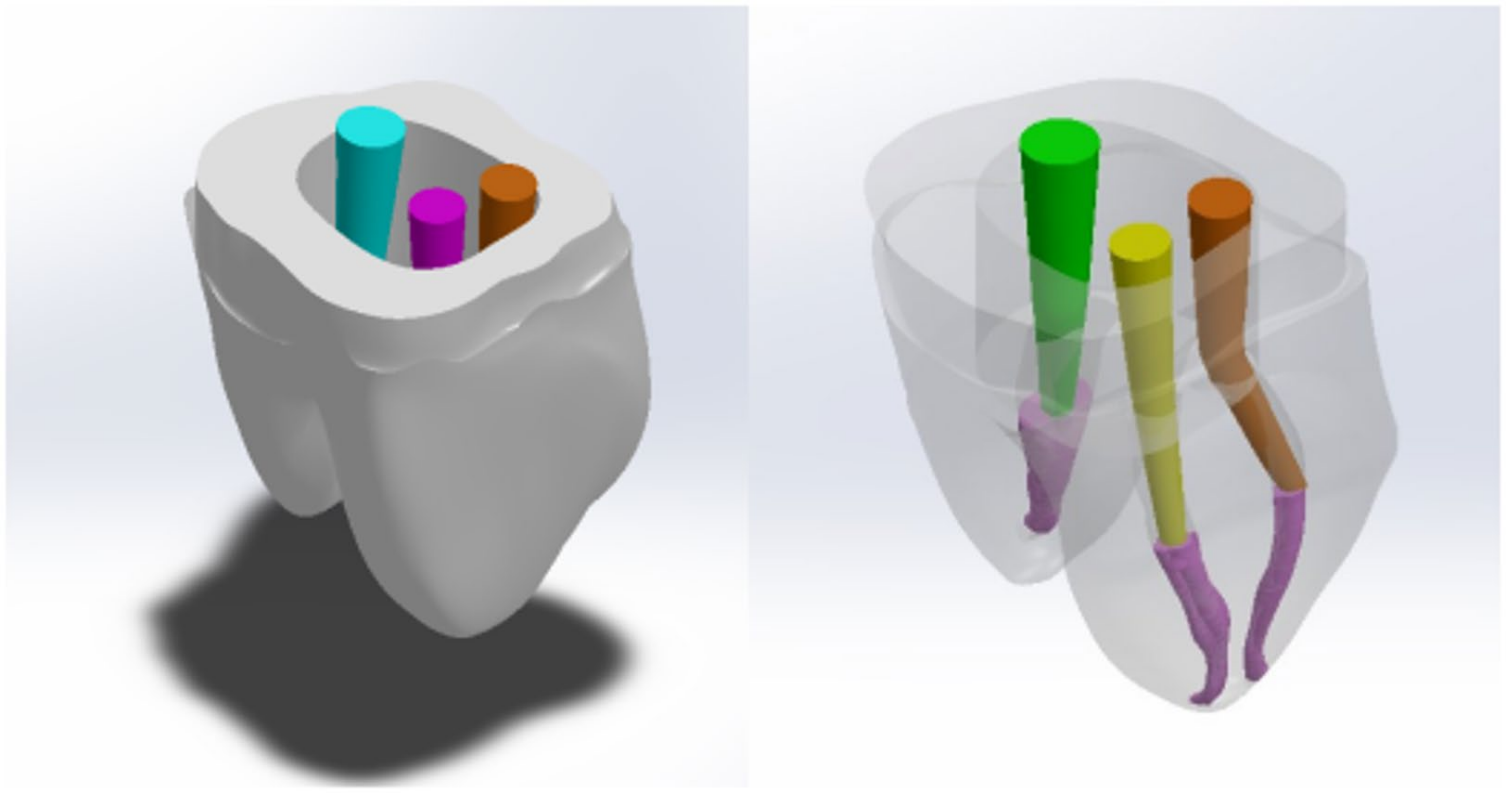
3D Mandibular right first molar model with posts inserted. Distal post,  Mesiobuccal post, Mesiolingual post.

The model was modified to simulate the clinical procedures, including access opening, root canal shaping, and filling. A traditional access cavity was created, removing the entire roof of the pulp chamber to provide a straight-line path from the access opening to the root canal orifice. The coronal portion was reduced to involve the occlusal two-thirds of the tooth, leaving 2.0 mm of tooth structure coronal to the CEJ, simulating a severely broken tooth with a ferrule effect. All three canals were shaped to a centralized conical shape, following the dimensions of the Protaper Next (PTN) rotary filing system (Dentsply; Maillefer, Ballaigues, Switzerland) with an estimated size X2: 25/0.06 taper. The apical third of the canals was obturated using gutta-percha points size X2 of the same PTN system, along with Bioceramic sealer (Brasseler USA, Savannah, GA) starting 0.5 mm from the apex and extending coronally for 5 mm. (Fig. 3)

Post space preparation was held using FILPOST Universal Groover held by a slow-speed handpiece performing a slow circular motion. Three posts of two different sizes were modeled and inserted into the root canals according to the manufacturer’s instructions. The coronal part of each post was bent to be parallel to the tooth axis and covered with a 0.4 mm layer of light-body material to represent the adhesive layer. The mesiobuccal and mesiolingual canals measured 1.3 mm in diameter, while the distal canal measured 2.0 mm. All gutta-percha was removed, leaving 5 mm of gutta-percha from the apex. The drilling length was 7.0 mm for the mesiobuccal and mesiolingual canals, and 8.0 mm for the distal canal from the CEJ, with a 1 mm layer of dentine thickness surrounding the post. A titanium post (FILPOST – ENDODONTIC ROOT CANAL POST) (Filhol Dental, Leamington Spa, U.K.) of 1.3 mm diameter and 17.5 mm length was inserted into the mesiobuccal and mesiolingual prepared canals, while a 1.65 mm diameter and 22.5 mm length post was inserted into the distal prepared canal.

The missing coronal tooth structure was restored with composite resin core material, maintaining 2 mm of dentine height to represent the ferrule. The tooth model was then prepared to receive a zirconia crown using the natural tooth crown shape with a 0.5 mm chamfer finish line and 1.5 mm occlusal reduction. (Fig. 3)

### Assembling models

Three model combinations were assembled as follows:

- Model D: Only distal canal post inserted.
- Model DMB: Distal canal post and mesiobuccal canal Post inserted.
- Model DML: Distal canal post and mesiolingual canal Post inserted. (Table 1).

**Table 1:** Locations of inserted posts in the three tested models.

Model	Distal canal post	Mesiobuccal canal post	Mesiolingual canal post
Model D	X		
Model DMB	X	X	
Model DML	X		X

#### Boundary conditions and load application

**Fig. 4:**
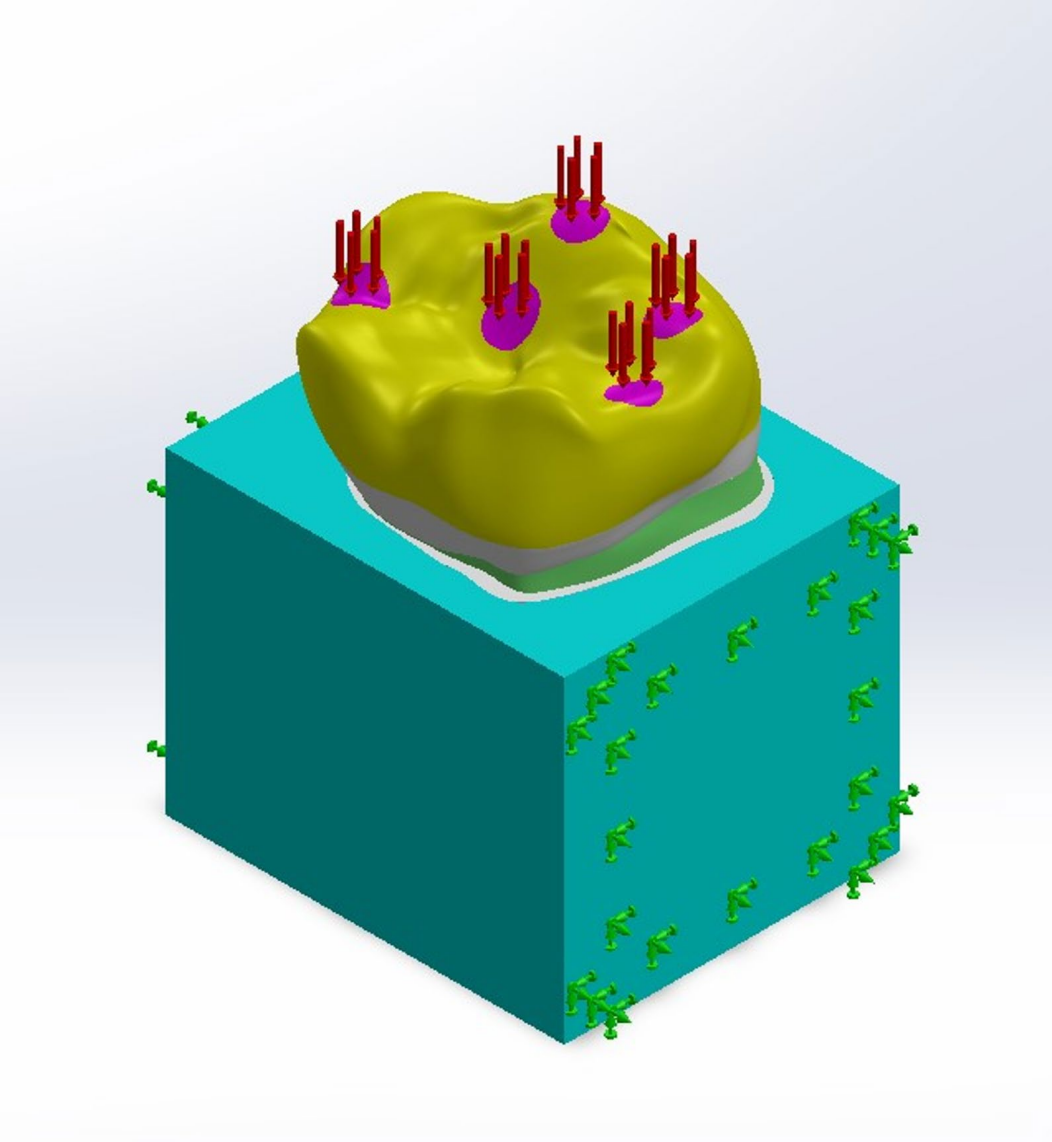
Boundary conditions and load application.

All bodies were simulated to be bonded to restrict the movement of a specific node or group of nodes within a finite element model. This constraint simulates a real-world situation where a physical object is fixed or attached to a stationary surface or structure, preventing it from moving freely in one or more directions. Boundary bonding means that no relative motion is permitted between the contacting surfaces of the two bodies. The cancellous bone block was fixed at both mesial and distal surfaces to isolate the ROI “Region of Interest” from outer effects. (Fig. 4)

A vertical compression force of 300 N was applied perpendicular to the occlusal plane at five 1 mm^2^ areas on the occlusal surface of the crown: mesiobuccal cusp, disto-buccal cusp, central fossa, mesial marginal ridge, and distal marginal ridge) (Fig. 4)^16^.

**Fig. 5:**
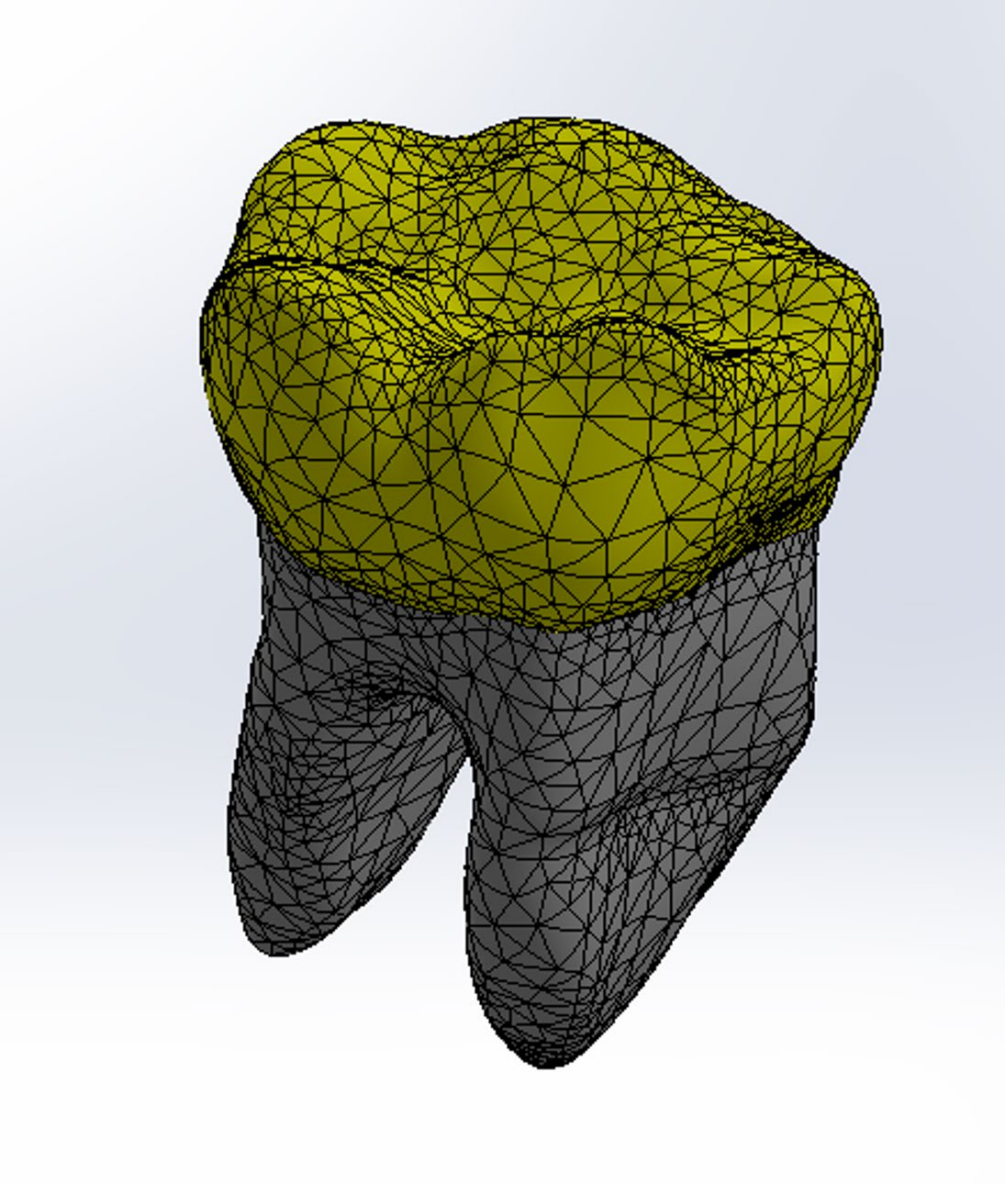
Mesh of the models.

The three models were meshed with six degrees of freedom per node: (Fig. 5)

- For Model D: 148,172 quadratic tetrahedral elements and 266,855 nodes.
- For Model DMB: 140,623 quadratic tetrahedral elements and 248,448 nodes.
- For Model DML: 151,075 quadratic tetrahedral elements and 271,295 nodes.

**Fig. 6:**
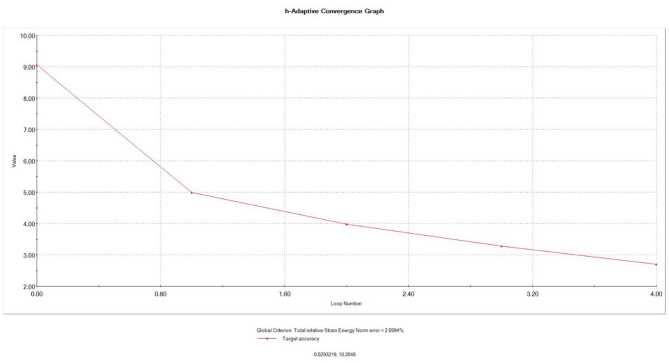
Mesh h-adaptive convergence graph.

The behavior of the meshed models during stress application as real situation was validated by mesh convergence analysis to calculate the error percentage to reduce the error to minimum acceptable which was considered 3% in our study. (Fig. 6)

The material properties used in the FEA models, including the elastic modulus and Poisson’s ratio dentin, cementum, cancellous bone, periodontal ligament, gutta-percha, titanium post, composite resin, adhesive resin cement, and zirconia crown are detailed in Table 2^16^.

**Table 2:** Elastic moduli and poisson’s ratio of the materials used in the 3D finite element model.

Material	Elastic modulus (GPa)	Poisson’s ratio
Dentin	18.6	0.31
Cementum	8.2	0.3
Cancellous bone	1.37	0.3
Periodontal ligament	0.05	0.45
Gutta percha	0.14	0.45
Titanium post	120	0.30
Composite resin	16.60	0.24
Adhesive resin cement	18.6	0.28
Zirconia	200	0.33

### Finite element analysis (FEA)

The 3D models of the lower mandibular molar were transferred to static structural analysis in ANSYS software (ANSYS Inc., Canonsburg, Pennsylvania, USA). A vertical compressive load of 300 N was applied perpendicular to the occlusal plane at five points on the occlusal surface (mesiobuccal cusp, disto-buccal cusp, central fossa, mesial marginal ridge, and distal marginal ridge). Stress distribution was represented as color-coded stress maps, with adjustable color scales corresponding to stress magnitude comparisons among the preparation designs for each analyzed structure. Von-Mises’s stress (MPa) values for the entire tooth–restoration assembly was recorded for the occlusal surfaces, including cusp tips and marginal ridge areas. Stress concentrations along the root canal length were also measured at different levels (7 mm, 5 mm, and 3 mm from the root apex)^16^.

## Results

### Occlusal surface stress distribution

Model D reported the highest von Mises stress values at occlusal surface (49.8 MPa). While Model DML exhibited the von Mises stress values (39.9 MPa) among the three Models. Model DMB displayed an intermediate von Mises stress values of 41.2 MPa. (Table 3)

### Finish line stress distribution

Model D reported the highest von Mises stress values at finish line Sect. (18 MPa). While Model DML exhibited the least von Mises stress values (11.2 MPa) among the three models. Model DMB displayed an intermediate von Mises stress values of 15.4 MPa. (Table 3)

### Furcation area stress distribution

Model D reported the highest von Mises stress values at furcation area Sect. (6.23 MPa). While Model DML exhibited the least von Mises stress values (1.9 MPa) among the three Models. Model DMB displayed an intermediate von Mises stress values of 2.93 MPa. (Table 3)

In each group separately, occlusal surface von Mises stress values were the highest reported stresses, followed by stresses at the finish line. While stress concentration at furcation area was minimum.

**Table 3.**
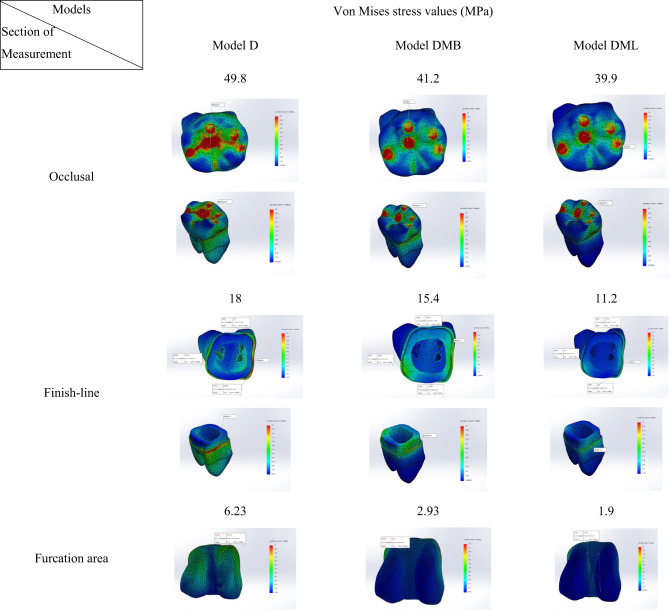
Comparison between maximum von Mises stress values (MPa) in the three models at different sections.

### Root sections stress distribution

Von Mises stress values along the root canal length were measured at three levels: cervical Sect. (7 mm from the apex), middle Sect. (5 mm from the apex), and apical Sect. (3 mm from the apex).

#### Cervical Sect. (7 mm from apex)

Model D had the highest von Mises stress values at the cervical section, with a value of 7 MPa. Model DML showed the least von Mises stress values of 1 MPa, while Model DMB had an intermediate von Mises stress values at this level (3.5 MPa). (Table 4)

#### Middle Sect. (5 mm from apex)

Model D had the highest stress concentration at the middle section, with a value of 6.5 MPa. Model DML showed the least stress concentration of 0.3 MPa, while Model DMB had an intermediate stress concentration at this level, with a value of 2.7 MPa. (Table 4)

#### Apical Sect. (3 mm from apex)

Model D had the highest stress concentration at the apical section, with a value of 6 MPa. Model DML showed the least stress concentration of 0.07 MPa, while Model DMB had an intermediate stress concentration at this level, with a value of 1.9 MPa. (Table 4)

For each individual group, stress concentration at the cervical section of the root was the maximum stresses exerted on the root surface. Then, stresses became diminished till reaching the apical section.

**Table 4.**
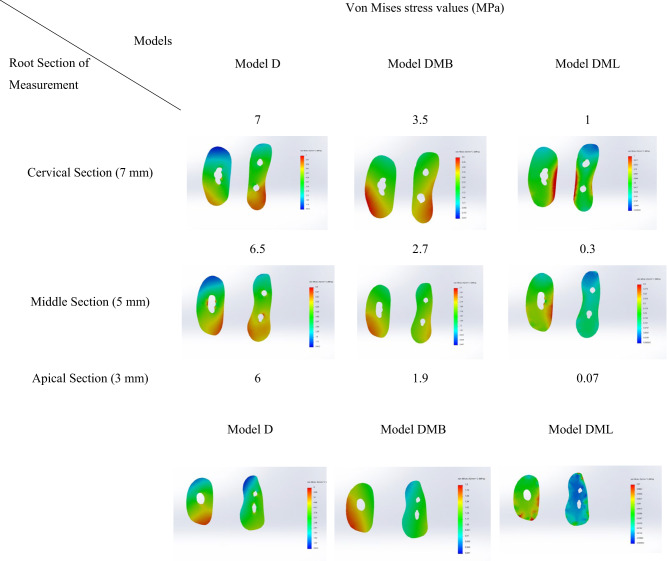
Comparison between von Mises stress values (MPa) in the three models at different root sections.

## Discussion

The effectiveness of ETT restoration hinges largely on the quality of the final restorations and how they are retained. This is especially crucial when significant tooth structure is missing, as it reduces the tooth’s resistance to chewing forces. The amount of healthy structure remaining, the properties of the post material, and the restoration’s design all influence the chosen restoration type^19^.

While numerous in-vitro studies have been conducted, the optimal post system, particularly regarding material selection, remains uncertain. Some researchers advocate for posts with a high modulus of elasticity, while others suggest a modulus closer to that of dentin. Additionally, there’s disagreement on whether a significant difference exists between these two approaches^20^.

In the present study, titanium based FILPOST has been used as it is engineered to be easier to place, even in difficult cases as curved canals, in a faster and safer manner. There is more preserving of healthy tooth structure, and it is stronger in use via its unique passive ‘interlocking’ system. FILPOST can be bent and shortened to suit the canal without risk of fracture, enabling easy insertion of multiple posts into converging canals^10^.

Singh SV et al.^1^ found that stresses in the dentin were almost similar when the carbon fiber post was compared to titanium post. However, stresses in the post and the cement were much higher when titanium post was used as compared to carbon fiber post. Similar results were reported by Nokar S et al.^8^ which was among the prefabricated post models, stainless steel, titanium, and zirconia posts demonstrated nearly the same stress levels between the middle and cervical thirds of the root. However, these three posts showed lower levels of VMS between the middle and cervical thirds of the root in comparison with FRC posts.

Panavia F self-adhesive resin cement was the selected cement in the current study for post-cementation, because it has an elasticity modulus that is comparable to dentin, which helps to improve stress distribution and strengthen the tooth structure^21^.

While laboratory tests provide valuable data on tooth fracture, they often involve destructive methods and cannot fully explore the stress-strain relationship of tooth restorations^22^. FEA is a powerful non-destructive tool in dental research for assessing stress distribution and mechanical behavior in dental structures. By creating a geometric model and dividing it into smaller elements, FEA uses mathematical equations to simulate the structure’s response to various forces. This method offers high accuracy and can model complex structures, including teeth and their supporting tissues, in great detail. 3D-FEA is a widely used numerical approach that simulates mechanical behavior based on material properties^23–26^. However, it’s important to remember that FEA cannot account for other factors that can affect restoration performance, such as microleakage, shrinkage, and sensitivity^27^.

Dental tissues exhibit anisotropic properties; however, isotropic models can produce comparable macro-scale stress and displacement patterns under clinically relevant loading conditions^28^. As, their coefficient of elasticity shows little differences in different directions under the applied force^29^. Likewise, the periodontal ligament demonstrates a small difference in stress distribution between linear and nonlinear response to force^30^. So, the current study assumed isotropic, linear elastic, and homogenous materials as an accepted simplification like other studies^31–33^. Interfaces were assumed to be fully bonded, with no gaps or voids.

Evaluating von Mises stresses for the whole assembly provides a comprehensive understanding of how occlusal loads are transferred and dissipated across the tooth–restoration complex. This approach reflects the clinical reality where the biomechanical performance of a restored tooth depends on the integrated response of dentin, post, core, and crown rather than any single component. Whole-assembly stress mapping allows identification of critical stress concentrations not only in dentin but also in restorative and post materials, which can influence the onset and location of failure. Analyzing the global stress field better predicts potential failure modes, as localized stresses in one material can result from load transfer from adjacent components^28^.

The results of the current study demonstrated that using multiple posts (Model DMB and Model DML) reported lesser values of stress in all measured areas, including the occlusal surface, finish line, furcation area, and along the root canal length when compared to using a single distal post (Model D) which exhibited the highest stress in all measured areas. This suggests that multiple posts can better distribute occlusal forces and enhance the mechanical performance of ETT.

The results of this study indicated that the number and position of posts impact stress distribution in ETT. Specifically, Model DML showed the lowest recorded stress values, suggesting that this configuration may provide the best mechanical performance and longevity for ETT. The mesial and distal root canals display different stress transmission characteristics, mainly because of a difference in morphology and mesiolingual angulation of the tooth^16^.

Coinciding results were reported by Zhong et al.^15^ who performed FEA on maxillary first molars with different post placements to analyze stress distribution. They found stress concentrations mainly at the cervical and furcation areas, with post location influencing stress in canals and interfaces. Vertical loading produced maximum stress in the palatal post, while lateral loading increased stress in buccal posts and interfaces. Overall, a single palatal post was most effective, with an additional mesiobuccal post improving lateral load resistance and retention.

Multiple studies, including those by Haralur^34^ and Frater^35^, have consistently found that using multiple fiber posts in dental restorations significantly improves fracture resistance compared to using a single fiber post. These studies used additional posts in the multipost technique while maintaining the same post space length in both groups.

Yoon HG et al.^16^ evaluated post location and occlusal load impact on stress distribution pattern inside the root canals of the mandibular 1 st molar. They found that the distal post model showed similar maximum stress values to the model with no post placement, while the mesiobuccal model showed markedly greater maximum stress values. They concluded that in the mandibular 1 st molar, the distal canal is the better place to insert the post in mesiobuccal canal only. However, if insertion into the mesiobuccal canal is unavoidable, there should be consideration on the occlusal contact, making central fossa and distal marginal ridge the main functioning areas.

A controversial study by Mayya and colleagues found that endodontically treated maxillary first premolars restored with either a single, longer post or two shorter, double posts exhibited similar fracture resistance. However, using two smaller posts can be a viable option for teeth with shorter, curved roots, as it helps preserve more tooth structure compared to using a single, larger post^9^.

Spicciarelli V. et al.^14^ found another conflicting result when comparing single and double posts in maxillary premolars. Their study suggested that for premolars with two separate roots, a single post in the palatal root canal might be a more conservative and safer option, simplifying potential future retreatment. The double-post technique did not offer additional fracture resistance in minimally damaged, endodontically treated premolars. Therefore, a single palatal post could be a more prudent choice.

Results of the current study showed that regarding stress values reported in each model separately, the occlusal surface stresses reported the highest stress value, followed by stresses at the finish line. While stress values at furcation area was minimum.

Contradictory finding was reported by Barcelos L et al.^13^ who observed that higher stress values were recorded at the furcation region of endodontically treated molar that should be considered during root canal preparation and during post space preparation. The preservation of dentin in this region is required for improving endodontically treated molar survival.

Regarding stresses along the three root sections, stress values were dramatically decreased to be the least at the apical section when two posts were used (Model DMB and Model DML). However, using single distal post subjecting the stresses measured at the three root sections to almost the same. Distal canal post model reported maximum stresses along the root surface sections rather than Model DMB and Model DML with two posts. This result could be explained by the influence of the straightness of the distal canal and the decrease of the circumference of root canal as it goes to the apex.

Likewise, Badami V. et al.^36^ reported maximum stress values at the cervical third of the root. When glass fiber posts were used. While prefabricated stainless steel and Titanium posts showed more stress values at the cervical and apical third of the root. Furthermore, prefabricated zirconia post showed maximum stress values at the middle third of the root. Controversial result was reported by the study of Liu, et al.^37^ in which they reported the stress increase at the apex and the decrease in the coronal root.

### Clinical implications

The results of this investigation have important therapeutic ramifications for ETT restoration. Multiple posts can enhance the mechanical performance and lifespan of restorations, especially in the distal and mesiolingual canals. By more equally distributing the occlusal stresses, this method lessens the chance of stress values and the ensuing fractures^15,34,35^.

### Limitations and future research

While this study provides valuable insights into stress distribution in ETT, it also has limitations. The FEA model used in this study is a simplification of the actual clinical scenario. Factors such as the presence of periodontal disease, variations in bone density, and patient-specific occlusal forces, micro-scale surface roughness of the post were not considered. Additionally, the simulated load of 300 N may not accurately represent the complex loading conditions experienced in the oral cavity such as fatigue loading^38^. Therefore, it is important to note that FEA cannot account for certain factors influencing restoration performance, including microleakage, polymerization shrinkage, and post-operative sensitivity^27^ and micro-scale surface topography of the post system and the root canal.

Furthermore, the simulated materials would present some defects that are not simulated in isotropic structures, there are, possible influences of oblique loading, sliding contacts and operator errors that are simplified^39^. Additionally, this study applied only a vertical compressive load, so the findings should be interpreted with caution, as real chewing forces often occur at an angle and could lead to different stress patterns or outcomes between the post designs.

Future research should focus on incorporating these variables into FEA models to provide a more comprehensive understanding of stress distribution in ETT. Clinical studies are also needed to validate the findings of this study and assess the long-term performance of different post designs and materials in real-world scenarios^40,41^. It would also be advantageous to investigate how fracture resistance and stress distribution are affected by various restorative materials and methods.

## Conclusions

The strategic placement of prefabricated posts in ETT mandibular first molars considerably influences stress distribution and the longevity of restorations. A double-post approach, particularly in the distal and mesiolingual canals, is optimal for dispersing occlusal forces and reducing stress concentrations. This approach enhances the mechanical integrity of the restoration, minimizing the risk of failure and prolonging the lifespan of multi-rooted posterior teeth.

Finite Element Analysis (FEA) has proven invaluable in predicting stress patterns and informing clinical decision-making in restorative dentistry with respecting to its limitation. Future research should focus on refining FEA models by incorporating clinically relevant oblique loading conditions and validating their findings through clinical trials to further optimize restoration outcomes for endodontically treated teeth.

## Supplementary Information

Below is the link to the electronic supplementary material.

Supplementary Material 1

## Data Availability

The original contributions presented in the study are included in the article. The STL file representing the complete 3D finite element model used in this study has been included as supplementary material. Further inquiries can be directed to the corresponding author.

## References

[1] Singh, S. V. et al. Stress distribution of endodontically treated teeth with titanium alloy post and carbon fiber post with different alveolar bone height: A three-dimensional finite element analysis. *Eur. J. Dent.* **9**, 428–432 (2015). 26430375 10.4103/1305-7456.163228 PMC4569998

[2] Dietschi, D., Duc, O., Krejci, I. & Sadan, A. Biomechanical considerations for the restoration of endodontically treated teeth: a systematic review of the literature–Part 1. Composition and micro- and macrostructure alterations. *Quintessence Int.* **38**, 733–743 (2007). 17873980

[3] Da Silva, P. B. et al. Influence of cervical preflaring and root Canal Preparation on the fracture resistance of endodontically treated teeth. *BMC Oral Health*. **20**, 111 (2020). 32299409 10.1186/s12903-020-1050-8 PMC7161170

[4] Nahar, R., Mishra, S. K. & Chowdhary, R. Evaluation of stress distribution in an endodontically treated tooth restored with four different post systems and two different crowns- a finite element analysis. *J. Oral Biol. Craniofac. Res.* **10**, 719–726 (2020). 33088703 10.1016/j.jobcr.2020.10.004 PMC7566941

[5] Singh, S. V. et al. Stress distribution of posts on the endodontically treated teeth with and without bone height augmentation: a three-dimensional finite element analysis. *J. Conserv. Dent.* **18**(3), 196–199 (2015). 26069403 10.4103/0972-0707.157242 PMC4450523

[6] De Carvalho, A. B. G. et al. Mechanical behavior of different restorative materials and onlay Preparation designs in endodontically treated molars. *Materials* **14**, 1923 (2021). 33921347 10.3390/ma14081923 PMC8070423

[7] Coelho, C. S. et al. Finite element analysis of weakened roots restored with composite resin and posts. *Dent. Mater. J.* **28**, 671–678 (2009). 20019417 10.4012/dmj.28.671

[8] Nokar, S., Bahrami, M. & Mostafavi, A. S. Comparative evaluation of the effect of different post and core materials on stress distribution in radicular dentin by Three-Dimensional finite element analysis. *J. Dent. (Tehran)*. **15** (2), 69–78 (2018). 29971124 PMC6026308

[9] Mayya, A., Naik, R., Mayya, S. S. & Paul, M. P. Fracture resistance of endodontically treated maxillary premolars with a longer single post and shorter double posts of different sizes: an *In vitro* study. *J. Int. Soc. Prev. Community Dent.* **10** (2), 183–184 (2020). 32670907 10.4103/jispcd.JISPCD_472_19 PMC7339994

[10] FILPOST restoration retention. system – better by design. *Br. Dent. J.* **221**, 44 (2016).

[11] Binus, S., Koch, A., Petschelt, A. & Berthold, C. Restoration of endodontically treated teeth with major hard tissue loss – bond strength of conventionally and adhesively luted fiber-reinforced composite posts. *Dent. Traumatol.* **29**, 339–354 (2013). 23171162 10.1111/edt.12013

[12] Lazari, P. C., de Carvalho, M. A., Del Bel Cury, A. A. & Magne, P. Survival of extensively damaged endodontically treated incisors restored with different types of posts-and-core foundation restoration material. *J. Prosthet. Dent.* **5**, 769–776 (2017). 10.1016/j.prosdent.2017.05.012 28923548

[13] Barcelos, L., Bicalho, A., Veríssimo, C., Rodrigues, M. & Soares, C. Stress distribution, tooth remaining strain, and fracture resistance of endodontically treated molars restored without or with one or two berglass posts and direct composite resin. *Oper. Dent.* **42** (6), 646–657 (2017). 28976843 10.2341/16-224-L

[14] Spicciarelli, V. et al. Different post placement strategies for the restoration of endodontically treated maxillary premolars with two roots: single post vs double post. *J. Contemp. Dent. Pract.* **21** (12), 1375 (2020). 33893261

[15] Zhong, Q. et al. Finite element analysis of maxillary first molar with a 4-wall defect and 1.5-mm-high ferrule restored with fiber-reinforced composite resin posts and resin core: the number and placement of the posts. *J. Prosthet. Dent.* **131**(1), 75–91 (2024). 35249741 10.1016/j.prosdent.2022.01.029

[16] Yoon, H. G., Oh, H. K., Lee, D. Y. & Shin, J. H. 3-D finite element analysis of the effects of post location and loading location on stress distribution in root canals of the mandibular 1st molar. *J. Appl. Oral Sci.* **26**, e20160406 (2018). 29451648 10.1590/1678-7757-2016-0406 PMC5815358

[17] Shetty, P. P., Meshramkar, R., Patil, K. N. & Nadiger, R. K. A finite element analysis for a comparative evaluation of stress with two commonly used esthetic posts. *Eur. J. Dent.* **7**, 419–422 (2013). 24932115 10.4103/1305-7456.120668 PMC4053665

[18] Rippe, M. P., Santini, M. F., Bier, C. A., Baldissara, P. & Valandro, L. F. Effect of root Canal preparation, type of endodontic post, and mechanical cycling on root fracture strength. *J. Appl. Oral Sci.* **22**, 165–173 (2014). 25025556 10.1590/1678-775720130051 PMC4072266

[19] Hazar, E. & Hazar, A. Effect of long glass fiber orientations or a Short-Fiber-Reinforced composite on the fracture resistance of endodontically treated premolars. *Polym. (Basel)*. **16** (9), 1289 (2024). 10.3390/polym16091289 PMC11085497 38732757

[20] Durmuş, G. & Oyar, P. Effects of post core materials on stress distribution in the restoration of mandibular second premolars: a finite element analysis. *J. Prosthet. Dent.* **112**, 547–554 (2014). 24630398 10.1016/j.prosdent.2013.12.006

[21] Narayanaswamy, S., Meena, N., Kumari, A. & Naveen, D. N. Finite element analysis of stress concentration in class V restorations of four groups of restorative materials in mandibular premolar. *J. Conserv. Dent.* **11**, 121–126 (2008). 20142899 10.4103/0972-0707.45251 PMC2813101

[22] Zarow, M. et al. Effect of Fiber Posts on Stress Distribution of Endodontically Treated Upper Premolars: Finite Element Analysis, Nanomaterials, Vol. 10, 1708 (2020). 10.3390/nano10091708 PMC7559636 32872519

[23] Cantó-Navés, O. et al. A 3D finite element analysis model of single Implant-Supported prosthesis under dynamic impact loading for evaluation of stress in the Crown, abutment and cortical bone using different rehabilitation materials. *Materials* **14**, 3519 (2021). 34202625 10.3390/ma14133519 PMC8269525

[24] Lee, J. H., Jang, H. Y. & Lee, S. Y. Finite element analysis of dental implants with zirconia crown restorations: conventional Cement-Retained vs. *Cementless Screw-Retained Mater.* **14**, 2666 (2021). 10.3390/ma14102666 PMC8160992 34069608

[25] Cicciù, M. Bioengineering methods of analysis and medical devices: A current trends and state of the Art. *Materials* **13**, 797 (2020). 32050530 10.3390/ma13030797 PMC7040794

[26] Nabih, S. M., Ibrahim, N. I. M. & Elmanakhly, A. R. Mechanical and thermal stress analysis of hybrid ceramic and lithium disilicate based ceramic CAD-CAM inlays using 3-D finite element analysis. *Braz Dent. Sci.* **24**, 1–10 (2021).

[27] Ausiello, P. et al. Effect of shrinking and no shrinking dentine and enamel replacing materials in posterior restoration: A 3D-FEA study. *Appl. Sci.* **11**, 2215 (2021).

[28] Aslan, T., Esim, E. & Üstün, Y. Finite element evaluation of dentin stress changes following different endodontic surgical approaches. *Odontology* **112** (3), 798–810 (2024). 38184512 10.1007/s10266-023-00882-1 PMC11269338

[29] González-Lluch, C., Pérez-González, A., Sancho-Bru, J. L. & Rodríguez-Cervantes, P. J. Mechanical performance of endodontic restorations with prefabricated posts: sensitivity analysis of parameters with a 3D finite element model. *Comput. Methods Biomech. Biomed. Engin*. **17**, 1108–1118 (2014). 23148761 10.1080/10255842.2012.737459

[30] Maceri, F., Martignoni, M. & Vairo, G. Optimal mechanical design of anatomical post-systems for endodontic restoration. *Comput. Methods Biomech. Biomed. Engin*. **12**, 59–71 (2009). 18629740 10.1080/10255840903065530

[31] de Andrade, G. S. et al. A study on stress distribution to cement layer and root dentin for post and cores made of CAD/CAM materials with different elasticity modulus in the absence of ferrule. *J. Clin. Exp. Dent.* **11**, 0–8 (2019). 10.4317/jced.55295 PMC6343998 30697387

[32] Marghalani, T. Y., Hamed, M. T., Awad, M. A., Naguib, G. H. & Elragi, A. F. Three-dimensional finite element analysis of custom-made ceramic dowel made using CAD/CAM technology. *J. Prosthodont.* **21**, 440–450 (2012). 22672191 10.1111/j.1532-849X.2012.00860.x

[33] Dal Piva, A. M., Tribst, J. P. & Souza, R. O. Borges AL influence of alveolar bone loss and cement layer thickness on the Biomechanical behavior of endodontically treated maxillary incisors: a 3-dimensional finite element analysis. *J. Endod*. **43**, 791–795 (2017). 28343925 10.1016/j.joen.2016.11.020

[34] Haralur, S. B., Al Ahmari, M. A., AlQarni, S. A. & Althobati, M. K. The effect of intraradicular multiple fiber and cast posts on the fracture resistance of endodontically treated teeth with wide root canals. *Biomed. Res. Int.* **2018**, 1671498 (2018). 30186851 10.1155/2018/1671498 PMC6114070

[35] Fráter, M. et al. In vitro fracture resistance of premolar teeth restored with fibre-reinforced composite posts using a single or a multi-post technique. *Aust Endod J.* **43**, 16–22 (2017). 27150658 10.1111/aej.12150

[36] Badami, V. et al. Comparative evaluation of different post materials on stress distribution in endodontically treated teeth using the finite element analysis method: A systematic review. *Cureus* **14** (9), e29753 (2022). 36324349 10.7759/cureus.29753 PMC9617588

[37] Liu, S., Liu, Y., Xu, J., Rong, Q. & Pan, S. Influence of occlusal contact and cusp inclination on the Biomechanical character of a maxillary premolar: a finite element analysis. *J. Prosthet. Dent.* **112**, 1238–1245 (2014). 24836532 10.1016/j.prosdent.2014.04.011

[38] Matuda, A. G. N. et al. Computer aided design modelling and finite element analysis of premolar proximal cavities restored with resin composites. *Materials* **14**, 2366 (2021). 34062936 10.3390/ma14092366 PMC8125402

[39] Tribst, J. P. M. et al. Survival Probability, Weibull Characteristics, stress Distribution, and fractographic analysis of Polymer-Infiltrated ceramic network restorations cemented on a chairside titanium base: an in vitro and in Silico study. *Materials* **13**, 1879 (2020). 32316360 10.3390/ma13081879 PMC7216243

[40] Boschian Pest, L., Guidotti, S., Pietrabissa, R. & Gagliani, M. Stress distribution in a post-restored tooth using the three-dimensional finite element method. *J. Oral Rehabil*. **33**, 690–697 (2006). 16922743 10.1111/j.1365-2842.2006.01538.x

[41] Benazzi, S., Grosse, I. R., Gruppioni, G., Weber, G. W. & Kullmer, O. Comparison of occlusal loading conditions in a lower second premolar using three-dimensional finite element analysis. *Clin. Oral Investig*. **18**, 369–375 (2014). 23504207 10.1007/s00784-013-0973-8

